# Apicomplexan actin polymerization depends on nucleation

**DOI:** 10.1038/s41598-017-11330-w

**Published:** 2017-09-22

**Authors:** Esa-Pekka Kumpula, Isa Pires, Devaki Lasiwa, Henni Piirainen, Ulrich Bergmann, Juha Vahokoski, Inari Kursula

**Affiliations:** 1 Biocenter Oulu and Faculty of Biochemistry and Molecular Medicine, University of Oulu, Aapistie 7, 90220 Oulu, Finland; 2 Department of Biomedicine, University of Bergen, Jonas Lies vei 91, 5009 Bergen, Norway

## Abstract

Filamentous actin is critical for apicomplexan motility and host cell invasion. Yet, parasite actin filaments are short and unstable. Their kinetic characterization has been hampered by the lack of robust quantitative methods. Using a modified labeling method, we carried out thorough biochemical characterization of malaria parasite actin. In contrast to the isodesmic polymerization mechanism suggested for *Toxoplasma gondii* actin, *Plasmodium falciparum* actin I polymerizes *via* the classical nucleation-elongation pathway, with kinetics similar to canonical actins. A high fragmentation rate, governed by weak lateral contacts within the filament, is likely the main reason for the short filament length. At steady state, *Plasmodium* actin is present in equal amounts of short filaments and dimers, with a small proportion of monomers, representing the apparent critical concentration of ~0.1 µM. The dimers polymerize but do not serve as nuclei. Our work enhances understanding of actin evolution and the mechanistic details of parasite motility, serving as a basis for exploring parasite actin and actin nucleators as drug targets against malaria and other apicomplexan parasitic diseases.

## Introduction

Actin is an extensively studied filament-forming cytoskeletal protein with roles ranging from endocytosis to vesicle trafficking and cell motility. Actin polymerizes by a nucleation-elongation mechanism, meaning that the process is split into a thermodynamically unfavorable nucleation phase, a linear elongation phase, and a plateau phase, where the rates of polymerization and depolymerization are equal. The rate-limiting step is nucleation, during which small nuclei, likely trimers^1–3^, form spontaneously and initiate the directional rapid polymerization from the fast-growing barbed end of the filament. The formation of nuclei and subsequent polymerization proceed only above a certain critical concentration (Cc) of actin, which in standard assay conditions is ~0.1 µM^4^.

*Plasmodium* spp. are unicellular protozoan parasites of the apicomplexan phylum and cause one of the globally most devastating human diseases: malaria. In the motile stages of the *Plasmodium* life cycle, the parasite moves by an active process called gliding, a form of motility unique to *Apicomplexa*. Gliding motility and host cell invasion rely on an unconventional actin-myosin motor^5^. The major actin isoform I, expressed also in the motile and invasive stages of the parasite, is one of the most divergent actin orthologs known^6–8^. *Plasmodium falciparum* actin I (*Pf*ActI) has a comparably low sequence identity to canonical actins (82% compared to human actins, 79% compared to *Saccharomyces cerevisiae* actin) and forms only short, transient filaments *in vitro* ^6,7,9^. Recently, it was proposed that actin of *Toxoplasma gondii*, a related apicomplexan parasite, polymerizes in an isodesmic manner that is not dependent on nucleation^10^. The isodesmic mechanism is characterized by a constant free-energy change at all stages of filament formation and exhibits therefore no lag phase and no Cc for polymerization^11^. Furthermore, the short length of apicomplexan actin filaments has been linked to unusually fast turnover^12^.

In *Plasmodium*, actin dynamics are regulated by a compact set of 10–15 actin-binding proteins^5^. *In vitro* characterization of these has been mainly based on interactions with canonical actins, due to the lack of biochemical means for measuring parasite actin polymerization kinetics reliably. Many of the most accurate methods available for analyzing actin kinetics *in vitro* rely on fluorescent labels. Conventional protocols of actin labeling employ cycles of polymerization and depolymerization to obtain a polymerization-competent pool of labeled actin. While this purification by biological activity increases the quality of the labeled protein preparation, the amount of sample and time consumed for the process is incompatible with the properties of apicomplexan actins^6,7^. We present here a simple, fast, and generally applicable protocol for labeling *Plasmodium* actin I with N-(1-pyrene)iodoacetamide (hereafter pyrene), and use fluorescence spectroscopy together with dynamic light scattering (DLS), native polyacrylamide gel electrophoresis (PAGE), and ultracentrifugation to analyze the biochemical properties of this so far kinetically uncharacterized actin.

## Results

### Polymerized *Pf*ActI is present in three different states of assembly at the steady state

**Figure 1:**
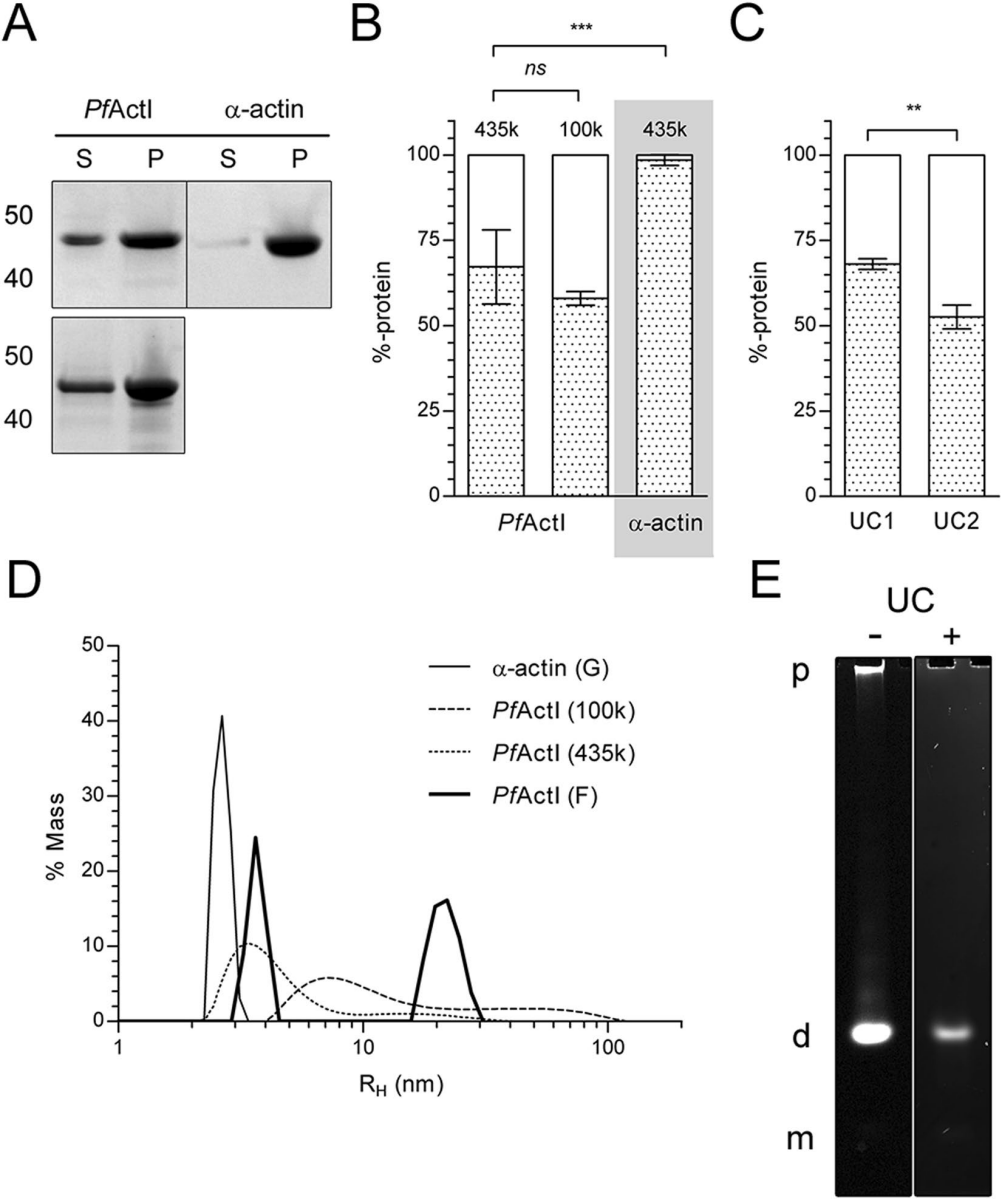
Assembly states of polymerized *Pf*ActI. (**A**) Representative gel of a standard pelleting assay of *Pf*ActI and α-actin at a total protein concentration of 4 µM, separated at 434,500 *g* (top) or 100,000 *g* (bottom) at 20 °C for 1 h. S and P stand for supernatant and pellet, respectively. Molecular weight standard sizes (in kDa) are indicated on the left. (**B**) Pelleting assay results of *Pf*ActI at 434,500 *g* (n = 12), at 100,000 *g* (n = 3), and α-actin at 434,500 *g* (n = 8), expressed as % of total protein in each fraction. Relative centrifugal forces have been indicated above each bar. (**C**) Two sequential pelleting assays (UC1 and UC2) of 10 µM *Pf*ActI, separated by 6 h of incubation at 22 °C, spun at 434,500 *g* for 1 h (n = 3). (**D**) DLS profiles of α-actin in the monomeric state, polymerized *Pf*ActI, and polymerized *Pf*ActI after ultracentrifugation at 100,000 *g* and 434,500 *g*. (**E**) Native PAGE of 25 µM polymerized *Pf*ActI before (left) and after (right) ultracentrifugation at 434,500 *g* in running conditions with sample-matched ATP:ADP ratio and 0.1 mM MgCl_2_. The sample after ultracentrifugation represents the supernatant. Letters on the left indicate monomers (m), dimers (d), or polymers (p), based on relative mobility values obtained before^7^. Images in (**E**) have been lightly contrast-adjusted. Error bars in (**B**) and (**C**) represent standard deviation, ***p < 0.001, **p < 0.05, *ns*: not significant, Student’s t-test.

To investigate the polymerization of *Pf*ActI, we first applied a high relative centrifugal force of 434,500 *g* in an attempt to pellet all forms of the short filaments. Even at these forces, *Pf*ActI pelleted less efficiently than skeletal muscle α-actin (70% compared to 99%; Fig. 1A,B and Supplementary Fig. S1) and exhibited higher standard deviations between batches (10.8% compared to 1.5%). Notably, approximately half of *Pf*ActI in the supernatant fraction could be re-pelleted 6 h after the initial ultracentrifugation step (Fig. 1C). This implies that the material in the supernatant is polymerization competent and is in line with the notion that the average filament length is concentration independent^13^.

As a substantial fraction of *Pf*ActI did not pellet during ultracentrifugation, we employed dynamic light scattering (DLS) to measure the size distribution of *Pf*ActI in polymerized samples. Polymerized *Pf*ActI showed a rough distribution into two populations with apparent hydrodynamic radii of 4.2 and 18 nm with mass percentage contributions of 35% and 65%, respectively. In terms of size, one can compare these to the hydrodynamic radius of α-actin in the G-state (2.7 nm, Fig. 1D) to infer that the size of the smaller population is, on average, larger than a monomer. Hydrodynamic radii of α-actin monomers as well as lateral and longitudinal dimers, constructed from high resolution filament models (PDB entry 3J8A^14^) and modeled using HYDROPRO^15^, are 3.4, 4.4, and 4.6 nm, respectively, placing the smaller population in the monomer-dimer domain. We also measured the size distribution of different oligomeric species of *Pf*ActI in the supernatant fraction after centrifugation at 434,500 *g*. We found a marked decrease in the mass contribution of the larger peak from 65% to 11%, but no change in the hydrodynamic radius of either peak (Fig. 1D). We also analyzed the samples at a lower centrifugal force of 100,000 *g*, where only 60% of *Pf*ActI was present in the pellet fraction (Fig. 1A,B). The supernatant fraction showed peaks in DLS at hydrodynamic radii of 10.5 and 53.6 nm, with 77% and 23% of total mass, respectively (Fig. 1D).

To confirm our observations in DLS measurements, we used native PAGE, which can resolve oligomers at least up to 12 units^7^. We prepared samples of polymerized *Pf*ActI at 25 µM concentration with and without ultracentrifugation at 434,500 *g* and analyzed them in running conditions with a matched ADP to ATP concentration ratio and 0.1 mM MgCl_2_ (Fig. 1E). In these conditions and without ultracentrifugation, 43% of *Pf*ActI appears polymeric, while the rest appears as a dimer band and an extremely weak monomer band (Supplementary Fig. S1). Ultracentrifugation removes the polymeric material but retains the dimer and monomer bands (Supplementary Fig. S1).

Collectively, these results show that *Pf*ActI is present as three distinct populations in a polymerized sample at the steady state: a lower molecular weight population with an average hydrodynamic radius of 4.2 nm, comprising monomers and dimers, and a higher molecular weight population at 18 nm. Furthermore, due to the abundance of oligomers too small to sediment even at high centrifugal forces, ultracentrifugation is not the method of choice for determining the Cc of apicomplexan actins.

### *Pf*ActI exhibits a critical concentration similar to canonical actins

We next determined, whether *Pf*ActI polymerizes in an isodesmic manner, as suggested for *T. gondii* actin^10^, or rather depends on a Cc like canonical actins. Indeed, when we analyzed the ultracentrifugation results of *Pf*ActI at different concentrations, as described before for *T. gondii* actin^10^, the data showed an absence of a plateau in the supernatant fraction (Supplementary Fig. S1), which could be interpreted as a lack of a Cc. The x-intercept of a linear fit of the pellet fraction, used in some studies^16^ as an indicator of a Cc from these plots, exhibited values ranging from 5 to 100 nM in identical conditions. Thus, we decided to use a more sensitive and reliable fluorescence spectroscopic method^17^ requiring pyrene-labeled actin. As labeling *Pf*ActI with pyrene at the Cys374 position was unsuccessful using established protocols, we modified the protocol to be applicable to parasite actins. Three essential modifications were necessary for labeling *Pf*ActI: (i) the removal of polymerization-depolymerization cycling, (ii) the use of substoichiometric concentrations of pyrene, and (iii) the use of very short reaction times. These modifications enabled a sufficient degree of labeling for several types of classical actin assays.

We verified the labeling of *Pf*ActI by liquid chromatography-coupled mass spectrometry (LC-MS) and compared the results to α-actin labeled with traditional approaches and our method (Supplementary Fig. S2). As another control, we analyzed α-actin labeled using both methods in a spontaneous polymerization assay (Supplementary Fig. S2). LC-MS indicated that α-actin was labeled at one site per monomer with both methods and *Pf*ActI at one site per monomer with the new method. The kinetic profiles of α-actin were different between the two methods, with the traditional labeling producing twice as fast overall polymerization compared to the new method and a different curve shape in the nucleation phase. Removing residual unreacted reactants by small-scale desalting did not change the results (Supplementary Fig. S2). A possible explanation for the different behavior is that the polymerization-depolymerization cycling in the classical method removes any monomers that may be inactivated during the labeling procedure. Another possibility is the formation of a small number of nucleating oligomers also in α-actin during the longer time required for the traditional labeling method. To allow direct comparison between the two different actins, we have used α-actin labeled with our method in all experiments.

**Figure 2:**
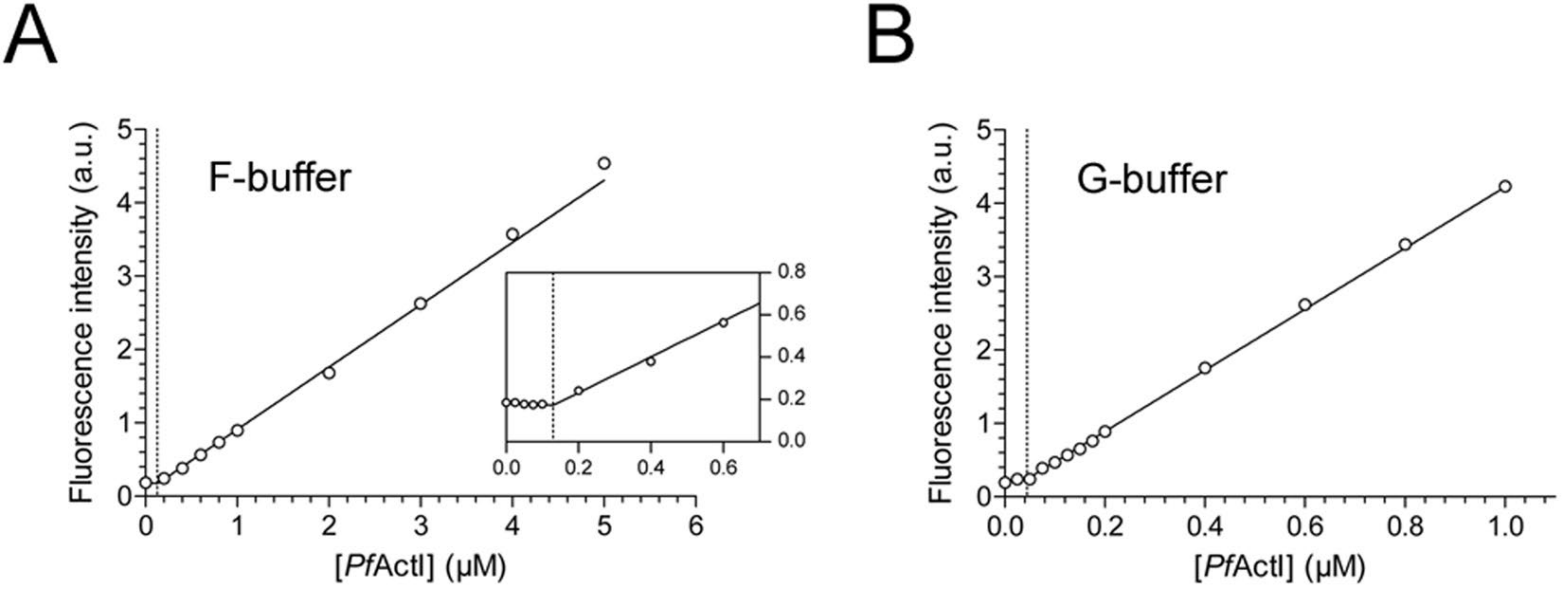
Critical concentration of *Pf*ActI determined using pyrene fluorescence. (**A**) Cc plot in F-buffer shows a critical concentration at 0.13 µM (indicated by the vertical dotted line). Points below 0.2 µM are excluded for clarity but shown in the inset. *Inset*: zoomed view of the lower concentrations. Axes of the inset are the same as in the full image. (**B**) Cc plot in G-buffer shows a critical concentration of 0.04 µM in this experiment (indicated by the vertical dotted line). Error bars representing standard deviation are hidden under the symbols. a.u. = arbitrary units, not comparable between (**A**) and (**B**). To analyze the data, a two-line fit was performed, as described in Materials and Methods.

The Cc of *Pf*ActI at steady state could be determined after polymerization for 16 h at 20 °C at different starting concentrations. A linear increase in fluorescence signal could be seen, starting at around 0.1 µM and extending to 5 µM, which was the highest concentration tested (Fig. 2A). Linear fits for the points below and above 0.1 µM were performed, and the Cc was determined as the x-coordinate of the intercept of these lines. A Cc of 0.11 ± 0.03 µM was found (mean ± standard deviation, n = 3). In the same conditions, α-actin exhibited a Cc of 0.06 ± 0.03 µM, which is slightly lower but statistically indistinguishable from the value obtained for *Pf*ActI (p = 0.18, Student’s t-test). Immediately after ultracentrifugation of pyrene-labeled polymerized *Pf*ActI, there was still fluorescence signal in the supernatant, confirming the presence of *Pf*ActI in non-monomeric, yet non-pelleting assemblies (Supplementary Fig. S2). Additionally, densitometry analysis of native PAGE (Fig. 1E, Supplementary Fig. S1) showed that 0.43% of total band intensity is present in the very weak monomer band. This translates into 0.11 µM in the 25 µM sample, which notably is identical to the Cc obtained at steady state. The rest of the band intensity was divided roughly equally between dimers (56.2%, 14 µM) and polymers (43.4%, 11 µM). The monomer and dimer bands are visible after ultracentrifugation, but the polymers and higher oligomers are not (Supplementary Fig. S1). Interestingly, *Pf*ActI incubated for 16 h at 20 °C in a classical non-polymerizing buffer also displayed a Cc of 0.06 ± 0.03 µM (mean ± standard deviation, n = 3) (Fig. 2B). This is in line with our previous observation that *Pf*ActI forms oligomers, likely upon ATP hydrolysis, in G-buffer conditions^7^.

### Spontaneous polymerization of *Pf*ActI is slow, yet depolymerization is fast

**Figure 3:**
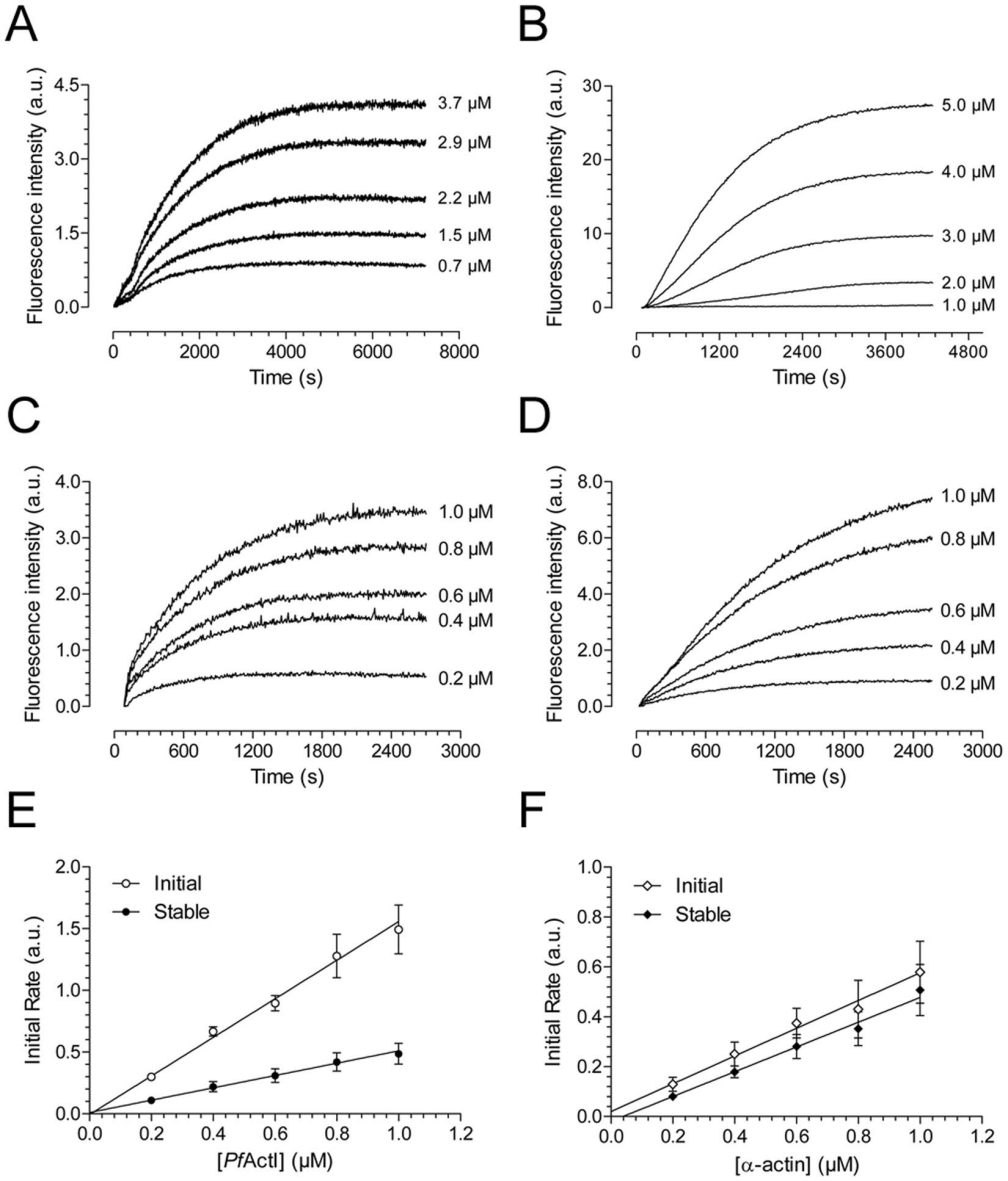
Polymerization kinetics of *Pf*ActI and α-actin as determined using pyrene fluorescence. (**A**) Spontaneous polymerization curves of *Pf*ActI induced by the addition of F-buffer components. (**B**) Spontaneous polymerization curves of α-actin as in (**A**). (**C**) Nucleated polymerization curves of *Pf*ActI seeded with 0.5 µM filamentous α-actin. (**D**) Nucleated polymerization curves of α-actin seeded with 0.5 µM filamentous α-actin. (**E**) Plot of elongation rates of *Pf*ActI from (**C**) *vs. Pf*ActI monomer concentration at the initial phase (0–120 s) and at the stable phase (60–600 s). (**F**) As in (**E**), but with α-actin. Pyrene labeling was performed using the fast labeling method for both *Pf*ActI and α-actin in all experiments. Note the different time scales in (**A**–**D**). a.u. = arbitrary units, scaled to achieve comparable rates between the two actins using plateau-level fluorescence.

After the discovery of a Cc for polymerization of *Pf*ActI, we decided to study its polymerization kinetics. However, spontaneous nucleation^7^ at the conditions used during purification and assays seemed initially to hinder recording of polymerization curves. A serendipitous discovery that relatively high concentrations of ammonium acetate could slow down the oligomerization of *Pf*ActI *in vitro*, as shown by DLS and native PAGE (Supplementary Fig. S3), led to a change in our initial protocol. Ammonium acetate also considerably slowed down α-actin nucleation (Supplementary Fig. S3). Inclusion of 0.3 M ammonium acetate in the final *Pf*ActI sample and removing it only immediately before the start of the measurement enabled us to record kinetic curves reproducibly (Fig. 3A). Still, the lag phase for *Pf*ActI polymerization was not as pronounced as for α-actin (Fig. 3B) and disappeared in the higher concentrations.

A critical kinetic parameter to determine for any actin is the elongation rate constant. As *Pf*ActI can co-polymerize with α-actin^6^ we determined elongation rates of *Pf*ActI in the presence of 0.5 µM polymerized α-actin over a range of monomer concentrations (Fig. 3C). The resulting curves were hyperbolic, and initial rates were determined and fitted against the monomeric *Pf*ActI concentration (Fig. 3E). Compared to similar data from α-actin (Fig. 3D,F), the curves of *Pf*ActI contained two distinct phases in the first few minutes, while those of α-actin contained only one. The slopes from the initial part (first 2 min) are significantly different (p < 0.01) between the two actins, while the slopes from the stable part (1–10 min) are statistically indistinguishable (Table 1). Notably, the estimations for Cc (x-intercept) and dissociation rate constant (y-intercept) are unreliable as exhibited by their large error estimates and sometimes incorrect signs for both α-actin and *Pf*ActI (Table 1). Combining the results from steady-state critical concentration and the slope of the curves in Fig. 3E,F, we can calculate a corresponding relative k_−_^*^. The resulting values are 0.05 ± 0.02 s^−1^ for *Pf*ActI and 0.03 ± 0.02 s^−1^ for α-actin (Table 1).

**Table 1:** Relative kinetic parameters of *Pf*ActI and α-actin.

	*Pf*ActI		α-actin	
	***Nucleated polymerization (NP)***			
	*Initial*	*Stable*	*Initial*	*Stable*
k_+_	1.56 ± 0.26^*^	0.50 ± 0.16	0.56 ± 0.16^*^	0.50 ± 0.10
Cc (µM)	0.00 ± 0.06	−0.02 ± 0.12	−0.04 ± 0.11	0.04 ± 0.07
k_−_	−0.01 ± 0.10	0.01 ± 0.06	0.02 ± 0.06	−0.02 ± 0.03
	***Steady state (SS)***			
Cc (µM)	0.11 ± 0.03		0.06 ± 0.03	
k_−_^†^	0.05 ± 0.02		0.03 ± 0.02	

All values reported as mean ± standard deviation (*Pf*ActI: n = 5 in NP, n = 3 in SS, α-actin: n = 3 in both NP and SS).

*p = 0.01 (two-tailed Student’s t-test)

^†^Relative k_−_ calculated using steady state Cc and the relative elongation rate constant (k_+_) from the stable phase of the nucleated polymerization assays.

**Figure 4:**
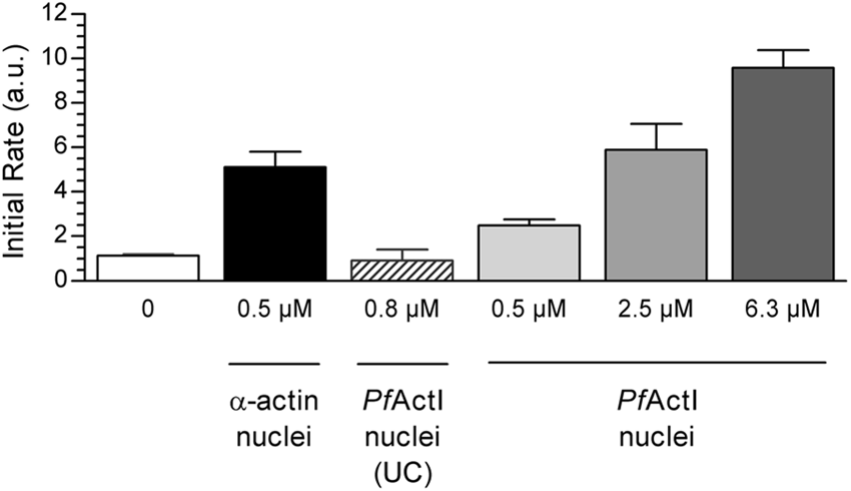
Nucleation of α-actin polymerization by *Pf*ActI filaments. 1 µM pyrene-labeled α-actin monomers were polymerized in the presence of 0.5 µM polymerized α-actin, the supernatant from a polymerized *Pf*ActI sample after ultracentrifugation at 434,500 *g* as well as 0.5 µM, 2.5 µM, and 6.25 µM polymerized *Pf*ActI. A linear fit of the first 2 min was performed on the resulting curves (Supplementary Fig. S4) and the slopes compared. Ultracentrifugation removes nucleating material from polymerized *Pf*ActI samples, and the nucleation efficiency of polymerized *Pf*ActI is lower towards α-actin than that of α-actin itself. Error bars represent standard deviation (n = 3). (a.u. = arbitrary units).

To assess nucleation of α-actin by *Pf*ActI filaments, we determined the initial rate of polymerization of pyrene labeled α-actin using α-actin filament seeds, three concentrations of polymerized *Pf*ActI, and the supernatant of ultracentrifuged *Pf*ActI filaments (Fig. 4, Supplementary Fig. S4). The results showed positive, but weaker nucleation of α-actin by *Pf*ActI filaments and a disappearance of nucleation activity upon removal of pelleting material by ultracentrifugation.

**Figure 5:**
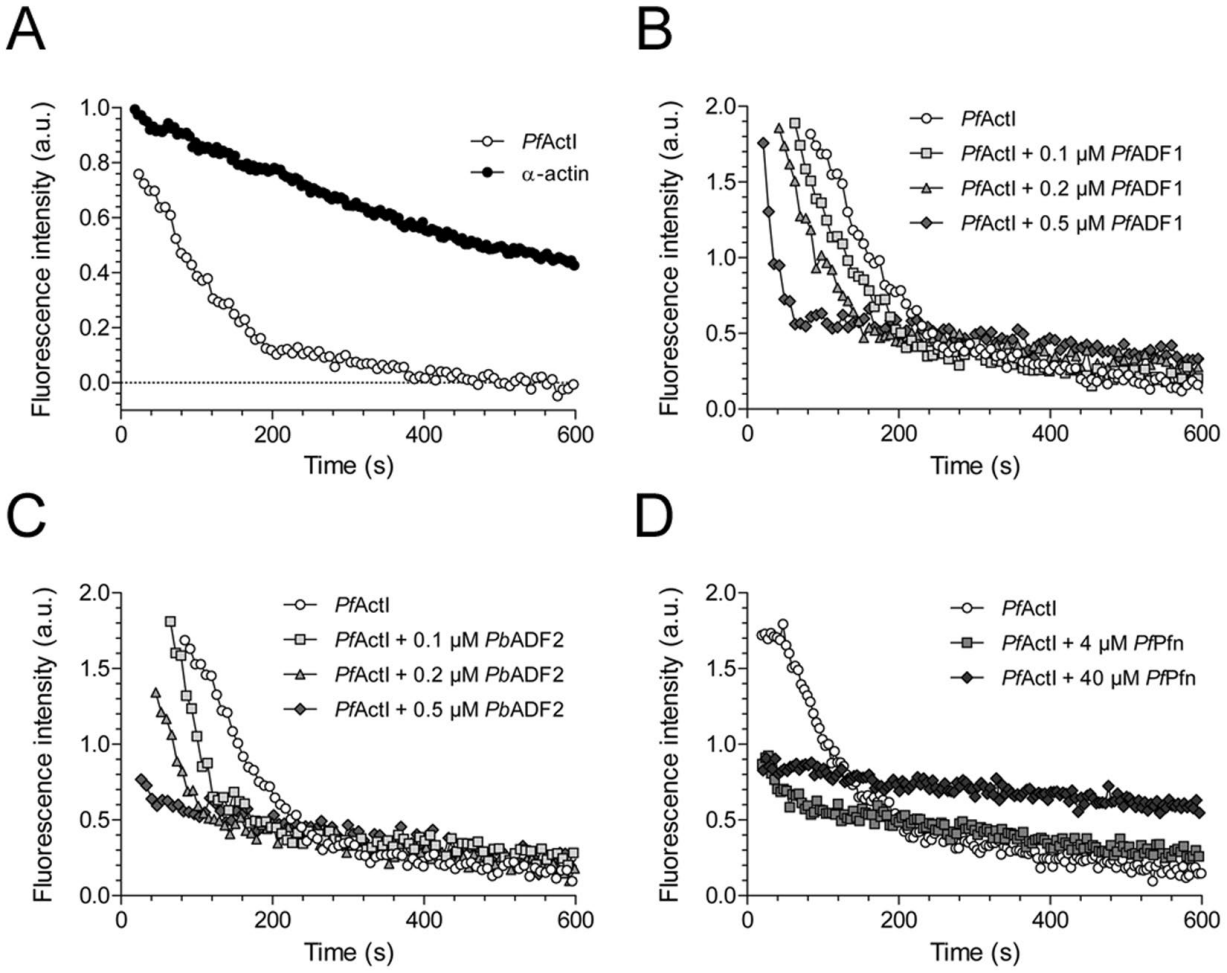
Dilution-induced depolymerization curves of *Pf*ActI. (**A**) Comparison of *Pf*ActI and α-actin, depolymerized by dilution from 5 µM to 50 nM. Half-times of depolymerization were 74 ± 4.3 s for *Pf*ActI (n = 3) and 376 ± 71 s for α-actin (n = 2). Values represent mean ± standard deviation. The rates are normalized based on a single-phase exponential decay fit of the data. (**B**) *Pf*ActI depolymerized in the presence of three concentrations of *Pf*ADF1 in the diluting buffer. For clarity, curves are moved to the right in increments of 20 s, leaving the curve with 0.5 µM *Pf*ADF1 at its initial place. (**C**) *Pf*ActI depolymerization as in (**B**), but supplemented with *Pb*ADF2 instead of *Pf*ADF1. (**D**) *Pf*ActI depolymerization in the presence of high concentrations of *Pf*Pfn in the dilution buffer. (a.u. = arbitrary units).

We next assayed the depolymerization of *Pf*ActI to determine, whether diluting the sample below the Cc would induce depolymerization. This was, indeed, the case, and *Pf*ActI depolymerized substantially faster than α-actin with half-times of 74 and 376 s, respectively (Fig. 5A). We then determined, whether the depolymerization of *Pf*ActI could be modulated by including recombinantly expressed *Plasmodium* actin-binding proteins. *P. falciparum* actin depolymerizing factor 1 (*Pf*ADF1) and *P. berghei* ADF2 (*Pb*ADF2) both increased the rate of depolymerization in a concentration-dependent manner (Fig. 5B,C). Depolymerization of gelsolin-capped filaments showed identical depolymerization curves to uncapped *Pf*ActI, even though we have previously shown that gelsolin segment 1 binds *Pf*ActI monomers^7^. *Pf*ADF1 affected both gelsolin-capped and uncapped filaments identically (Supplementary Fig. S4). Similarly to the parasite ADFs and human profilin^18^, high concentrations of *P. falciparum* profilin (*Pf*Pfn) increased the depolymerization rate as well as the final fluorescence level (Fig. 5D). These results strongly indicate that the folding of recombinant *Pf*ActI produced in baculovirus-infected insect cells is correct, as the biological interaction between *Pf*ActI and *Plasmodium* ADFs and profilin can be reconstituted *in vitro*. This, together with our previous functional characterization and high-resolution crystal structures of both *Plasmodium* actin isoforms^7,19,20^ and the high-resolution structure of *Pf*ActI in its F-form^21^ should remove any doubts about the folding state of recombinantly expressed *Pf*ActI raised by recent speculations^22^.

Treadmilling assays revealed that, while treadmilling of α-actin could be accelerated by *Pf*ADF1 as expected^23^, *Pf*ActI treadmilling appears unaffected by the inclusion of either *Pf*ADF1 or *Pb*ADF2 (Supplementary Fig. S4). In addition, the overall profile is different for the actins alone. This implies differences either in the effect of ADFs on *Pf*ActI treadmilling or in the way *Pf*ActI releases its bound nucleotide.

In conclusion, the *Pf*ActI elongation rate constant (k_+_) is statistically indistinguishable from α-actin, and its net depolymerization rate, as well as the apparent dissociation rate constant (k_−_^*^), is larger. This should lead to a higher Cc for *Pf*ActI than α-actin. However, although we do see a small difference in this direction in our experimental conditions, this difference is not statistically significant. In conditions nucleated by α-actin filaments, *Pf*ActI polymerizes faster initially but slows down to α-actin levels over a time span of a few minutes. Despite the faster depolymerization and shorter filament length, treadmilling of *Pf*ActI seems unexpectedly slow and, moreover, is not affected by the presence of otherwise active ADFs.

## Discussion

Established labeling protocols for actin rely on labeling in the filamentous state, combined with one or more cycles of polymerization and depolymerization^17,24^. However, sensitive actin samples may be either incompletely polymerizing, available in low quantities, unstable over time, or all of the above, as is the case for apicomplexan actins. For these cases, a fast labeling method requiring no polymerization-depolymerization cycling and low sample quantities is essential. To address these issues, we developed a method that allowed us to characterize the properties of *Pf*ActI that has so far eluded detailed kinetic characterization. In stark contrast to what has been reported for *T. gondii* actin based on sedimentation by ultracentrifugation^10^, the fluorescence spectroscopic assay shows that *Pf*ActI exhibits a Cc for polymerization at ~0.1 µM, which is similar to the behavior of canonical actins. A similar or slightly lower Cc in G-buffer is in line with our earlier observation that both *Pf*ActI and *Pb*ActII oligomerize in G-buffer over time and upon ATP hydrolysis^7^. The concomitant pyrene fluorescence signal increase upon the formation of these oligomers confirms that these oligomers are not an artefact of the native PAGE assay. Above the Cc, a major part of *Pf*ActI is split roughly equally to polymeric and dimeric components. After removal of the polymeric component by ultracentrifugation, the remaining component could not nucleate α-actin polymerization, demonstrating that the dimers cannot serve as nuclei. This is in agreement with reports on α-actin that propose the nucleus to be a trimer or tetramer^1–3,25^. Furthermore, plots of fluorescence *vs. Pf*ActI concentration (Supplementary Fig. S2) indicated that polymers disappear below the Cc.

*Pf*ActI filaments have been proposed to be highly dynamic, which implies fast treadmilling and, thus, fast on and off rates^26^. In contrast, we show that the elongation rate of α-actin-seeded *Pf*ActI relative to α-actin is statistically indistinguishable, which is in line with evidence that the elongation rate constant is diffusion-limited^27^. The initial fast phase of the nucleated polymerization - a 2.8-fold increase in slope compared to α-actin – could be explained by rapid formation of non-nucleating dimers and their subsequent incorporation into the filaments in the initial stages. Another possible scenario could stem from the heterologous interactions of α-actin barbed ends with *Pf*ActI monomer pointed ends, which could induce long-range changes in the filament conformation. However, it seems implausible that this alone could be responsible for an increase of the observed magnitude. In any case, the enhancement of overall polymerization rates by introduction of nuclei to *Pf*ActI supports the nucleation-elongation mechanism. Conversely, *Pf*ActI also nucleates α-actin polymerization. Taking into account that only 70% of *Pf*ActI is in a polymeric form results in an estimated 2.6-fold lower nucleation efficiency compared to α-actin. Combined, these results imply that interactions between α-actin barbed ends and *Pf*ActI monomers are favorable, while those of *Pf*ActI barbed ends and α-actin monomers are unfavorable. This is in line with many of the residue-level changes in *Pf*ActI being located at the pointed-end interface^7^. We assume that pointed ends of the nuclei or the low concentration of monomers in the nucleating solutions in these experiments do not contribute significantly to the observed rates.

Although not functioning as nuclei, the *Pf*ActI dimers are polymerization competent. There are two possibilities for the structural arrangement that pertain to what happens in a re-equilibrating supernatant fraction of *Pf*ActI: either the dimers are arranged in a conformation that facilitates polymerization directly or in a conformation that does not. In both cases, polymerization *via* dissociation of the dimers is possible. In the former case, annealing of dimers is also an option. As estimates of annealing rate constants for short filaments, of which dimers are an extreme case^28–30^, are comparable to the association rate constants at the barbed end, resolving this difference with simple kinetic assays is not straightforward. Considering that simulations suggest very high K_d_ values for dimer formation of α-actin^1,3,31,32^, the presence of a large pool of dimers in polymerized *Pf*ActI is interesting and probably reflects the weaker lateral interactions in the filament. The most probable route for nucleus formation is the formation of a longitudinal dimer, followed by the addition of a monomer to start the second protofilament^1^. Since interprotofilament contacts in *Pf*ActI filaments are among the more diverged from α-actin filaments^7^ and ostensibly facilitate weaker interactions, it is conceivable that the dimers are longitudinal rather than lateral, and the addition of the third protomer, *i.e*. nucleus formation is the rate limiting step, as in canonical actins. In fact, one would thus expect *Pf*ActI to be even more dependent on nucleation promoting factors compared to canonical actins.

Contrary to the polymerization rate, the overall depolymerization rate of *Pf*ActI is faster compared to α-actin. However, dilution-induced depolymerization curves are difficult to quantify, due to several overlapping decays contributing to the measured curves^33^, so only qualitative information can be extracted. Assuming a single-phase decay, the obtained half-times correspond to apparent rate constants of 9.3 × 10^–3^ s^−1^ for *Pf*ActI and 1.8 × 10^−3^ s^−1^ for α-actin. Adjusting by filament concentration determined from experiments in Figs 3, 4 and Supplementary Fig. S4 (α-actin^34^) or by assuming 100 nm filament length (*Pf*ActI) yields depolymerization rate constants of 7.2 s^−1^ and 0.4 s^−1^, respectively. On the other hand, the k_−_^*^ (Table 1) of *Pf*ActI is 2-fold larger than that of α-actin. We hypothesize that this discrepancy can be explained by a significant reduction of k_−_ of ADP-*Pf*ActI from the barbed end, which is the main determinant of depolymerization rate in our experimental conditions, but would not affect the kinetics of polymerizing ATP-*Pf*ActI. As an alternative explanation, substantial increases in filament length (1–2 µm) would be expected in order to raise the depolymerization rate constant to α-actin levels. Such long filaments of *Pf*ActI in the absence of stabilizing agents have not been documented.

*Pf*ADF1, *Pb*ADF2 and *Pf*Pfn seem to modulate the depolymerization of *Pf*ActI as expected. However, we cannot completely rule out fluorescence quenching upon filament binding as a cause for the observed increase in the apparent depolymerization rate. Yet, the increase in the final fluorescence level is rather indicative of monomer binding^35^. Capping *Pf*ActI filaments with human plasma gelsolin (Supplementary Fig. S4) did not change the effect of *Pf*ADF1, implying that the depolymerization activity of *Pf*ADF1 is located at the pointed end. Surprisingly, the apparent treadmilling rate of *Pf*ActI was not faster than that of α-actin, and unlike the depolymerization rate, the treadmilling rate was not affected by *Plasmodium* ADFs. This could be related to how *Pf*ActI interacts with ε-ATP, since we were previously unable to exchange the nucleotide of *Pf*ActI to non-hydrolyzable analogues^7^. On the other hand, the initial signals from α-actin and *Pf*ActI samples were similar, indicating incorporation of ε-ATP. *Pf*ADF1 increases the nucleotide exchange rate of bovine β-actin^36^, measured in the direction Ca-ATP to ε-ATP. Here, the direction is Mg-ε-ADP to ATP, which is a crucial difference. ADFs preferentially bind ADP-actin^23^, and the nucleotide exchange effect could be modulated by the nucleotide state and the bound cation.

## Concluding Remarks

In contrast to the isodesmic model suggested for the polymerization of *T. gondii* actin, we show that *Plasmodium falciparum* actin I polymerizes *via* the classical nucleation-elongation pathway. Taking into account the high sequence similarity between different apicomplexan actins and that the sedimentation-based methods are insensitive to short oligomers, it seems likely that also other parasitic actins follow a similar nucleation-elongation polymerization pathway. This is also supported by recent *in vivo* work on *T*. *gondii* actin^37,38^. Since the kinetic parameters of polymerization as well as treadmilling rates were similar in *Pf*ActI and α-actin, the observed differences in filament lengths would have to be due to changes in the fragmentation-annealing equilibrium. This is a defining factor for the steady-state filament lengths of α-actin^13,39^. Both processes are governed in *Pf*ActI by weaker interprotofilament contacts, which also likely explain the large dimer content at steady state. Thus, targeting the divergent lateral contacts may turn out to be a way to specifically inhibit parasite actin polymerization. Our data also suggest that nucleation is of particular importance for parasite actin-related processes. The small number of actin regulators, in particular only the formins present as nucleating factors, should make this a vulnerable step in the parasite life cycle. Thus, parasite formins, being also highly diverged from their opisthokont homologues, should be considered as drug targets against malaria and other apicomplexan parasitic diseases.

## Materials and Methods

### Protein expression and purification

Recombinant *Pf*ActI, *Pf*ADF1, *Pb*ADF2, and *P*fPfn were purified using standard protocols, as described before^40^ and in the Supplementary Methods. Skeletal muscle α-actin was purified from pig muscle as described before^40,41^.

### Pelleting assays

Unlabeled actin was polymerized overnight at 22 °C by adding F-buffer to a final polymerizing condition of 50 mM KCl, 4 mM MgCl_2_, 1 mM EGTA, followed by ultracentrifugation at 434,500 *g* at 20 °C for 1 h. The supernatant was removed and either prepared directly or after acetone precipitation for SDS-PAGE. The pellet from ultracentrifugation was resuspended directly into SDS-PAGE sample buffer in an identical volume to the corresponding supernatant sample. The samples were separated using SDS-PAGE in Tris-glycine buffer. When a large linear response range was desired (as in Supplementary Fig. S1), gels were stained by SYPRO Orange gel stain (Invitrogen) in 7.5% acetic acid overnight, washed for 30 s with 7.5% acetic acid, then transferred to ultrapure water and imaged using a BioRad ChemiDoc XRS+ gel imager. Alternatively, gels were stained with colloidal Coomassie (Thermo Scientific, 24620). The intensities of the corresponding supernatant and pellet bands were extracted using the program ImageLab (BioRad) and compared to their sum to get a fraction of total actin in each fraction and multiplying it by the total concentration of actin in the sample.

When determining the oligomeric state of actin in the supernatant fraction, samples of overnight polymerized actin were spun at either 100,000 *g* or 434,500 *g* for 1 h at 20 °C. The supernatant fraction was separated and analyzed by DLS, native PAGE and SDS-PAGE – the latter together with the pellet sample.

### Dynamic light scattering and native gel electrophoresis

DLS was measured using a Wyatt DynaPro PlateReader-II instrument in a 384-well plate (Corning, 3540). Long acquisition times (up to 60 s) were probed to record signals from longer filaments. Mass percentage contribution to individual peaks in the regularization graph was estimated using the coils model of the DYNAMICS program (version 7.1.7.16). The constraints for fitting were loosened by one from the resolution slider in the regularization graph options to resolve the peaks.

Native PAGE was performed essentially as described^7^. However, the ratio of ATP to ADP was determined from the steady-state samples prior to native PAGE using the ADP-Glo™ kit (Promega), and a corresponding mixture of ATP and ADP was used in the running buffer (see Supplementary Methods). Gels were stained with SYPRO Orange to improve sensitivity and with Coomassie brilliant blue in Supplementary Fig. S3.

### Critical concentration determination

Pyrene-labeled *Pf*ActI was polymerized by adding F-buffer, diluting to a series of concentrations, and incubating overnight at 22 °C. The fluorescence was then measured from triplicate samples using a fluorescence plate reader with identical optical settings as in the polymerization assays (see below). The sample volume was 200 µl. The results were fitted using a two-line equation:

$${\rm{y}}={{\rm{y}}}_{{\rm{cross}}}+({\rm{x}}-{{\rm{x}}}_{{\rm{cross}}})\times {\rm{k}}$$

where x_cross_ and y_cross_ are shared x and y coordinates for the two lines at their intersection and k is the slope. The fitting was performed using 1/Y^2^ weighting in GraphPad Prism 5.04.

### Actin polymerization assays

Polymerization assays were carried out in a TECAN M1000 Pro instrument using 96-well plates (Greiner 655077), an excitation wavelength of 365 nm (9 nm bandpass) and an emission wavelength of 407 nm (20 nm bandpass). The gain was set manually to 100 for all measurements and the number of flashes to 5 per measurement. Polymerization was initiated with F-buffer in a total reaction volume of 150 µl. Before the start of the measurement, a 2 s orbital mixing step was performed at 258 rpm (2.5 mm amplitude).

Nucleated polymerization assays were carried out using freshly labeled actin without dilution by unlabeled actin. Nuclei were prepared essentially as described before^34^, whereby an unlabeled filament stock of 5 µM was diluted to 0.75 µM in 1.5X F-buffer and vortexed for 0.5 s. These diluted filaments were immediately used for assays. A 100-µl aliquot of this mixture was carefully transferred into measurement wells before adding 50 µl of labeled actin monomers at three times the final concentration. Polymerization curves were recorded using the settings above. Nucleated polymerization assays with *Pf*ActI nuclei were prepared as above, using a constant 1.0 µM labeled α-actin monomer concentration. *Pf*ActI samples were pelleted at 434,500 *g* and used as nuclei immediately.

### Depolymerization assays

Dilution-induced depolymerization assays were performed using 30% pyrene-labeled actins. 5 µM actins were polymerized overnight as described above. Depolymerization was induced by diluting a 2-µl sample rapidly 100-fold in F-buffer containing ADFs or profilin, when applicable, and the measurement conducted as above for the polymerization assays, with the exception of not including a mixing step in the beginning to reduce dead times to a minimum.

### Treadmilling assays

Treadmilling assays were prepared essentially as described^17^ with the exception of using similar actin buffers and polymerizing conditions as used in our other experiments and polymerizing *Pf*ActI for 2 h. Briefly, DOWEX 1 × 8 200–400 (Sigma Aldrich) was used to remove free ATP from the actin solution, an excess of 1,*N* ^6^-ethenoadenosine 5′-triphosphate (ε-ATP, Jena Biosciences) was supplied for an overnight incubation on ice, followed by a removal of ε-ATP by DOWEX and adding a trace amount of ε-ATP. The Ca-ε-ATP:actin was then polymerized and the polymerized sample was treated with ATP to chase out the bound ε-ATP while the fluorescence was monitored over time. The fluorescence signal was read using an excitation wavelength of 350 nm (9 nm bandpass) and an emission of 410 nm (20 nm bandpass) using the same instrument as above.

### Data availability

The datasets generated and analyzed during the current study are available from the corresponding author upon reasonable request.

## Electronic supplementary material

Supplementary Information
