# Supplementary material for: Apicomplexan actin polymerization depends on nucleation
Source: Sci Rep. 2017 Sep 22;7:12137. doi: 10.1038/s41598-017-11330-w (PMC5610305; doi:10.1038/s41598-017-11330-w)
Supplement: Supplementary file 1 — Supplementary Information [file 41598_2017_11330_MOESM1_ESM.pdf]

SUPPLEMENTARY INFORMATION

Apicomplexan actin polymerization depends on nucleation

Esa-Pekka Kumpula, Isa Pires, Devaki Lasiwa, Henni Piirainen, Ulrich Bergmann, Juha Vahokoski & Inari Kursula

SUPPLEMENTARY METHODS

Protein expression and purification

N-terminally His-tagged *Pf*ActI was expressed as described previously[1,2]. All purification steps were performed either on ice or at 4°C, unless otherwise indicated. Cells pelleted in lysis buffer (10 mM HEPES pH 7.5, 5 mM $CaCl_2$, 250 mM NaCl, 15 mM imidazole pH 7.5, 7 mM β-mercaptoethanol, 1 mM ATP) were lysed by sonication. The clarified lysate was mixed with Ni-NTA equilibrated with lysis buffer with the β-mercaptoethanol concentration reduced to 3 mM for 1 h using gentle agitation. The matrix was washed with the same buffer, step-wise increasing the salt and imidazole concentrations to 500 and 25 mM, respectively, and finally with G-buffer (10 mM HEPES pH 7.5, 0.2 mM $CaCl_2$, 3 mM β-mercaptoethanol, 0.5 mM ATP). The protein was eluted with G-buffer supplemented with 300 mM imidazole and imidazole removed using a PD-10 column (GE Healthcare). The His-tag was cleaved with TEV protease either overnight in the presence of 300 mM ammonium acetate (pH 7.3) in G buffer or for 1 h at 20°C without ammonium acetate, and the tag and any uncleaved protein were removed using Ni-NTA. Concentrated samples were polished using size exclusion chromatography with either a Superose 12 10/300 GL (GE Healthcare) or Superdex 200 10/300 GL (GE Healthcare) column equilibrated with G-buffer with 0.5 mM TCEP. For samples used in polymerization kinetics, this buffer also contained 300 mM ammonium acetate.

*Pf*ADF1 and *Pb*ADF2 were expressed as N-terminally His-tagged constructs with a SUMO3 tag preceding the ADF sequence. *Pf*ADF1 was expressed in *Escherichia coli* BL21(DE3) RIPL and *Pb*ADF2 in *E. coli* Rosetta(DE3) cells for 36 h at 20°C in ZYM-5052 medium[3]. The *Pf*ADF1 pellet was resuspended in 20 mM Tris-Cl, pH 8.0, 50 mM NaCl, 5 mM β-mercaptoethanol, 5 mM

imidazole, 1X protease inhibitor cocktail (S8830, Sigma Aldrich), lysed by sonication, and purified using standard Ni-NTA procedures. The tag was cleaved using SENP2 protease[4] under dialysis against 20 mM HEPES pH 7.0, 50 mM NaCl, 0.5 mM TCEP. The tag and any uncleaved protein were removed using Ni-NTA, and the final protein was polished using a HiLoad 16/60 Superdex 75 column (GE Healthcare). *Pb*ADF2 was purified in an identical fashion, with the exception of 20 mM Tris-Cl pH 8.0 used as the buffering component in the dialysis and final gel filtration buffers. *Pf*Pfn was expressed and purified as described before[5].

ADP determination

Before analysis of polymerized *Pf*ActI by native PAGE, the concentration of ADP was determined using ADP-Glo™ (Promega) using the manufacturer's instructions. The final running buffer for the native PAGE assays was then supplemented with the determined concentration of ADP and ATP was added such that the final nucleotide concentration was 0.45 mM.

Pyrene labeling

The fast pyrene labeling was achieved by treating actin at a concentration of 10-20 µM with NPI at a ratio of 1:2 (NPI:actin) from a 2 mM stock solution in DMF for 30 s and stopping the reaction by adding DTT from a 0.1 M stock solution to a molar ratio of 10:1 to 20:1 (DTT:NPI). Approximately 30% of actin was labeled, based on absorbance at 344 nm after desalting. The labeled actin was diluted by unlabeled actin by 1/3, unless otherwise stated. For recording *Pf*ActI spontaneous polymerization curves, the sample was gel filtered with 300 mM ammonium acetate in the buffer, concentrated to 10-20 µM, and labeled with the fast pyrene labeling protocol using a 5-min reaction time instead of 30 s. The labeled protein was then run through a SpinTrap G-25 column (GE Healthcare) equilibrated with gel filtration buffer without ammonium acetate, immediately diluted to the correct concentration, and polymerized in the fluorescence plate reader. Skeletal muscle α-actin was labeled either using the fast pyrene label protocol and diluted with unlabeled actin to 10% label, or using established protocols[6,7].

As shown by Cc plots, spontaneous and nucleated polymerization assays, and dilution-induced depolymerization assays, this labeling method produces results comparable to those obtained by established labeling protocols, although kinetic timescales in spontaneous polymerization assays

showed some differences. Removal of labeling reaction components by small-scale desalting produced identical spontaneous polymerization curves compared to untreated samples, indicating that the labeling likely inactivates a portion of the monomers that in established protocols is removed during polymerization-depolymerization cycling.

Mass spectrometry data acquisition and processing

The molecular weights of the actin species were measured by ESI-LCMS using a Synapt G1 (Waters) Q-TOF instrument linked to an Acquity (Waters) UPLC chromatography system. The protein solution was diluted 10-fold with 0.1% trifluoroacetic acid (TFA) and 2 µl aliquots were injected into a Waters BEH300 C4 (1.7 µm, 2.1 × 100 mm) column. The samples were eluted with a gradient of 0.1% formic acid in water (A) and 0.1% formic acid in acetonitrile (B) from 0 to 60% B over a time course of 8 min. The source of the mass spectrometer was tuned with standard settings for the detection proteins and 1 s lock mass (Leu encephalin) corrected scans were measured in the mass range from 500 to 2000 m/z. Combined mass spectra for the chromatographic peaks were deconvoluted using the MaxEnt 1 algorithm as part of the instrument software (Masslynx).

SUPPORTING FIGURES

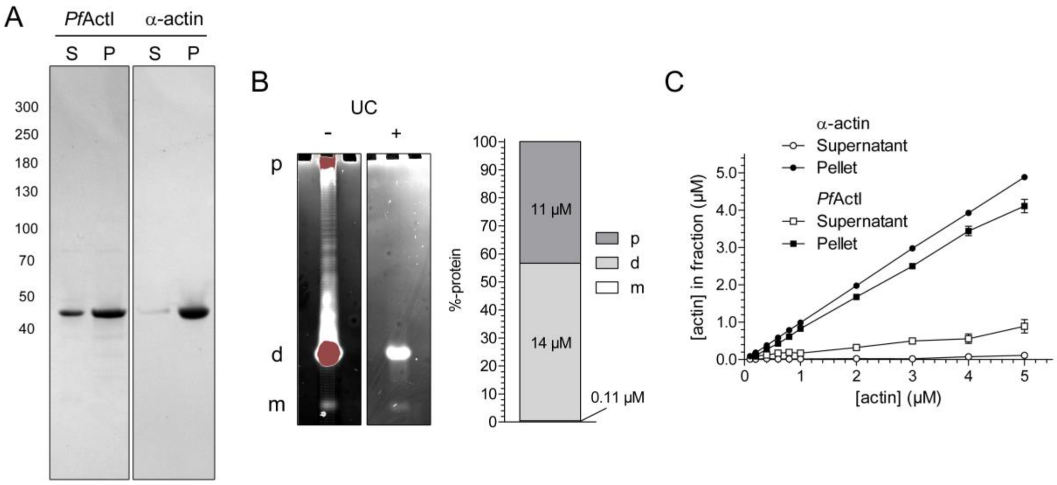

Fig. S1. Properties of unlabeled, polymerized *Pf*ActI. (A) Polymerized *Pf*ActI and α-actin were ultracentrifuged at 434,500 *g*, 20°C for 1 h at a concentration of 4 µM followed by SDS-PAGE analysis. The figure shows the entire lanes, parts of which are shown in **Fig. 1A**. Molecular weight standard positions are indicated on the left in kDa. S and P stand for supernatant and pellet, respectively. (B) Presence of monomer band in polymerized *Pf*ActI in native PAGE. *Left*: Polymerized *Pf*ActI at a concentration of 25 µM was run in native PAGE using a running buffer with sample-matched ATP:ADP ratio (0.35 mM ATP, 0.1 mM ADP; see methods) and 25 mM Tris-Cl, 125 mM glycine pH 8.5, 0.1 mM $MgCl_2$ either before or after ultracentrifugation at 434,500 *g*, 20°C for 1 h. This image (UC -) represents overexposed image of the lane in **Fig. 1E** on the left, highlighting the presence of the very weak monomer band. Red areas are overexposed and an acquisition artefact caused by the overexposed polymer band is visible throughout the lane. The ultracentrifuged sample (UC +) is the same image as in **Fig. 1E**, but the contrast has been enhanced to show the monomer band. The lowercase letters denote monomers (m), dimers (d) and polymers (p). *Right*: Distribution of *Pf*ActI in the three different populations evaluated by densitometry of the bands at two different exposure levels of the image. Intensities were scaled based on a dilute sample present in both exposure levels. Note that the monomer concentration derived from the fraction of total intensity is in a remarkably good agreement with the critical concentration derived from steady

state measurements (**Fig. 2A** and main text). (C) Critical concentration determination by pelleting assay. Polymerized *Pf*ActI and α-actin were analyzed by ultracentrifugation at 434,500 g, 20°C, 1 h, followed by SDS-PAGE, SYPRO Orange staining and densitometry of the supernatant and pellet fractions up to a concentration of 5 μM. Concentrations in each fraction were determined by comparing the intensity of the fraction to the total intensity of both fractions and multiplied by the total concentration in the original sample. α-actin is indicated by circles and *Pf*ActI by squares, while open and closed symbols represent supernatants and pellets, respectively.

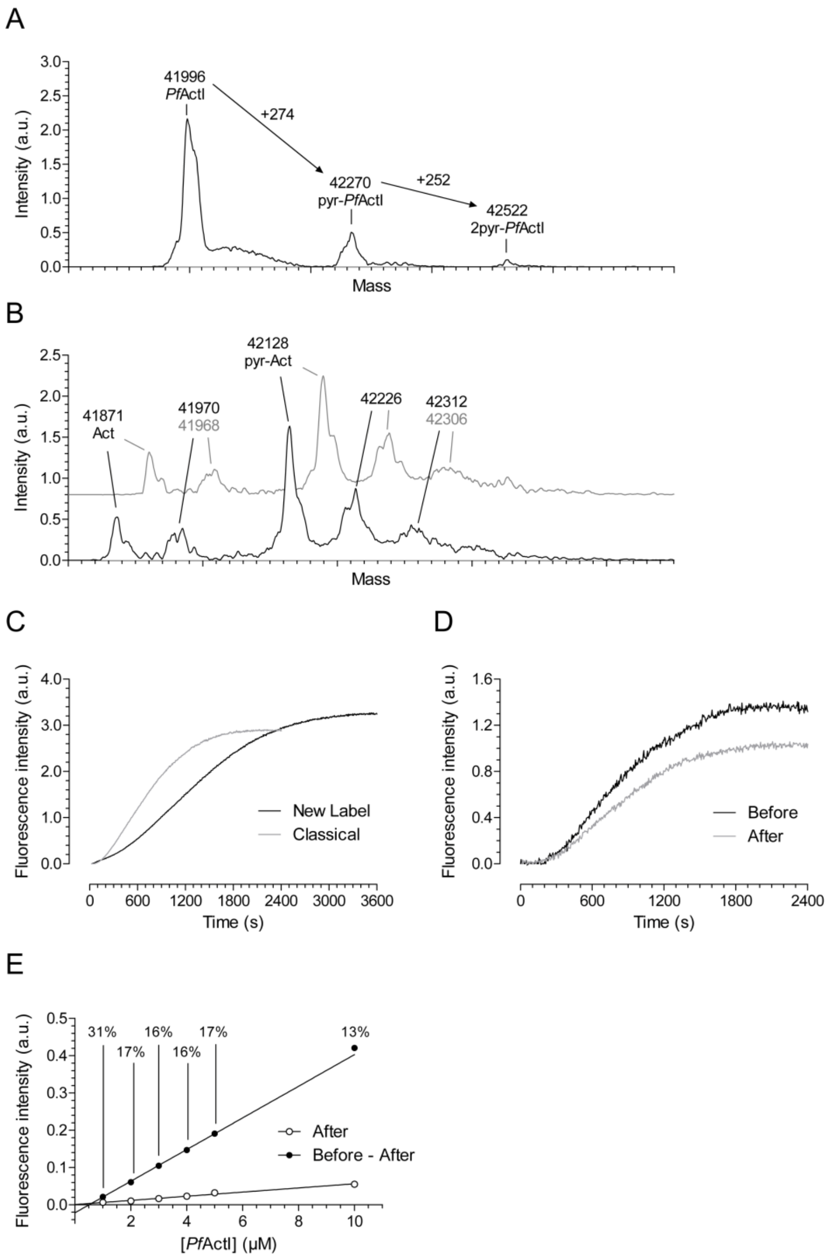

Fig. S2. Control experiments for the new pyrene labeling protocol and pyrene fluorescence of *Pf*ActI before and after ultracentrifugation. (A) LC-MS analysis of labeled *Pf*ActI. *Pf*ActI was labeled as described in Materials and Methods in the monomeric state without dilution with unlabeled *Pf*ActI and the labeled product was analyzed by LC-MS. Numbers above the peaks are average peak masses in daltons (Da) and the numbers above the arrows are Δm values in Da between the two peaks. The 41996 Da peak corresponds to the *Pf*ActI construct size with a theoretical molecular weight of 41998.77 Da (containing extra Gly-Ala in the N-terminus compared

to UniProt entry PFL2215w) without modifications, the 42270 Da peak to singly pyrene-labeled and oxidized *Pf*ActI and the 42522 peak to doubly pyrene-labeled and oxidized *Pf*ActI. The broad peak next to the 41996 Da peak is likely a deconvolution artefact. (B) LC-MS analysis of α-actin labeled with the new (black) and established (grey) methods. α-actin was labeled with an established protocol and the new method presented in this paper and the resulting samples were run in LC-MS as in (A). Numbers above the peaks correspond to average masses and are indicated in black only when the masses differ by < 1 Da and in both black and grey when the average masses differ by > 1 Da. The grey curve is shifted up and to the right to enhance visibility. The 41871 Da peak corresponds to methylated and N-terminally mature α-actin (N-terminal Met and Cys removed and the remaining N-terminus acetylated, His73 methylated, resulting in a theoretical average mass of 41872 Da) and the 42128 Da peak to singly pyrene labeled α-actin (Δm = 257 Da). Peaks 41968 Da, 41970 Da, 42226 Da, 42306 Da and 42312 Da (Δm values of 97 Da, 99 Da, 98 Da, 80 Da and 86 Da to the closest lower molecular weight peak, respectively) are possibly artefacts and roughly correspond to the size of single (41970 Da, 41968 Da, 42226 Da) or double (42312 Da, 42306 Da) TFA adducts. (C) Spontaneous polymerization assay results of α-actin labeled with the new (black) and established (grey) methods. Assays were carried out as in the materials and methods section, at a final protein concentration of 4 µM. (D) Lack of effect by the unreacted pyrene and other labeling components on α-actin polymerization demonstrated by spontaneous polymerization assays before (black) and after (grey) removal of contaminants using a small-scale desalting column. Polymerization assays were carried out at 4 µM concentration and at a low pyrene labeling percentage of 2.5%. The decrease in plateau level is caused by the loss of material in the desalting step. (E) Pyrene fluorescence of a series of *Pf*ActI concentrations was measured before and immediately after ultracentrifugation at 434,500 *g*. In this figure, the fluorescence of after and (before – after) are reported, which correspond to fluorescence of the supernatant and pellet, respectively. Linear regression showed x-intercepts for the data at -0.1 and 0.5 µM for the supernatant and pellet, respectively. Above are indicated the corresponding percentages of fluorescence contained at each concentration after ultracentrifugation compared to before. (a.u. = arbitrary units)

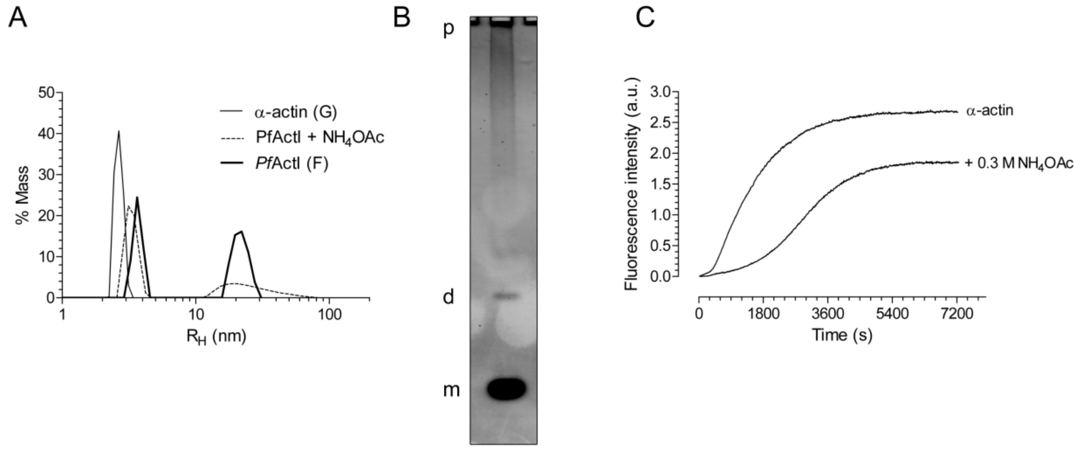

Fig. S3. Effect of ammonium acetate on the nucleation of *Pf*ActI and α-actin. (A) DLS of α-actin in the G-state (thin line), *Pf*ActI in F-state (thick line; polymerized for 16 h at 22°C) and *Pf*ActI stored for 5 d at 4°C in the presence of 0.3 M ammonium acetate demonstrates that over these timescales, ammonium acetate inhibits oligomerization and subsequent polymerization of *Pf*ActI. (B) Native PAGE of 5 µM *Pf*ActI stored for 7 d at 4°C in the presence of 0.3 M ammonium acetate demonstrates that most of *Pf*ActI is still monomeric after a long storage period in these conditions. (C) Spontaneous polymerization assay of 5 µM α-actin with and without 0.3 M ammonium acetate present in the polymerization reaction shows an extended lag phase and a reduced plateau level. (a.u. = arbitrary units)

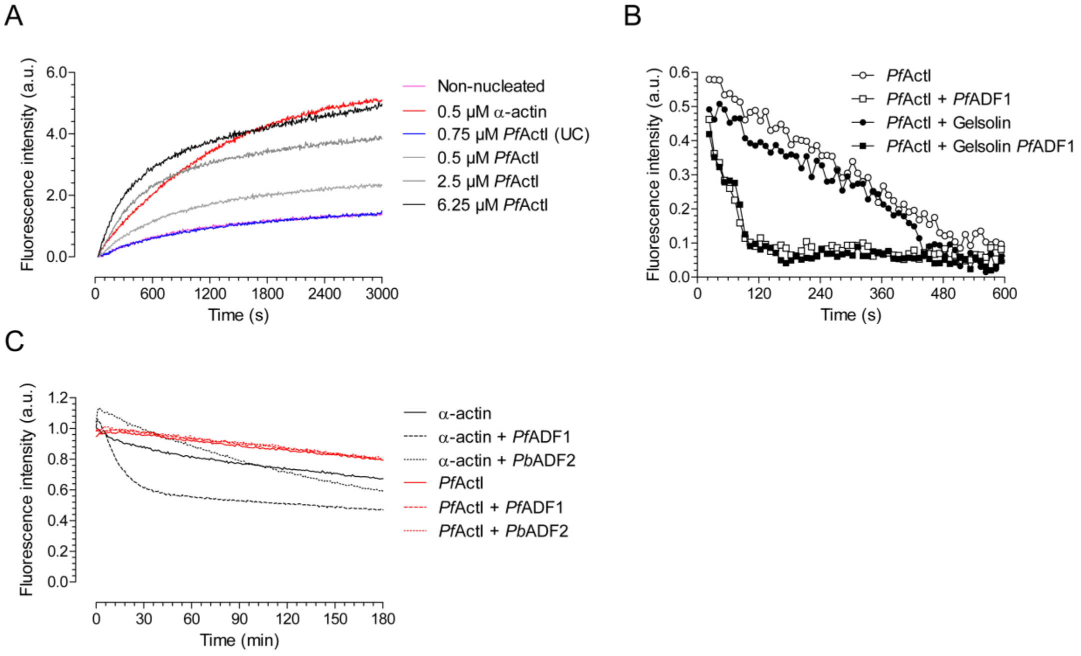

Fig. S4. Nucleation, treadmilling and gelsolin-capped depolymerization of *Pf*ActI. (A) Nucleated polymerization curves of 1 µM α-actin in the presence of no nuclei (pink), 0.5 µM polymerized α-actin (red), supernatant of 2.5 µM polymerized *Pf*ActI after ultracentrifugation in a final *Pf*ActI concentration of 0.75 µM (blue), 0.5 µM (light grey), 2.5 µM (grey) and 6.25 µM (black) polymerized *Pf*ActI. All concentrations reported are final concentrations of protein in the measured sample. (B) Depolymerization of *Pf*ActI (open circles) in the presence of either human plasma gelsolin (solid circles) or *Pf*ADF1 at 0.5 µM (open squares) or both (closed squares). Pyrene labeled *Pf*ActI was polymerized at a concentration of 5 µM and in the presence of 0.2 µM gelsolin (when needed) for 16 h at 20°C in F-buffer without EGTA. (C) Treadmilling assay results of 7 µM α-actin (black solid line) with 7 µM *Pf*ADF1 (black dashed line) or 7 µM *Pb*ADF2 (black dotted line), 7 µM *Pf*ActI (red solid line) with 7 µM *Pf*ADF1 (dashed red line) or 7 µM *Pb*ADF2 (red dotted line). Curves are normalized such that the intensity measured before the start of reaction is equal to 1.0. (a.u. = arbitrary units)

REFERENCES

1. Bhargav, S. P., Vahokoski, J., Kumpula, E.-P. & Kursula, I. Crystallization and preliminary structural characterization of the two actin isoforms of the malaria parasite. *Acta Crystallogr. Sect. F. Struct. Biol. Cryst. Commun.* **69,** 1171–1176 (2013).

2. Vahokoski, J. *et al.* Structural Differences Explain Diverse Functions of Plasmodium Actins. *PLoS Pathog.* **10,** e1004091 (2014).

3. Studier, F. W. Protein production by auto-induction in high density shaking cultures. *Protein Expr. Purif.* **41,** 207–234 (2005).

4. Reverter, D. & Lima, C. D. Preparation of SUMO proteases and kinetic analysis using endogenous substrates. *Methods Mol. Biol.* **497,** 225–239 (2009).

5. Ignatev, A., Bhargav, S. P., Vahokoski, J., Kursula, P. & Kursula, I. The lasso segment is required for functional dimerization of the Plasmodium formin 1 FH2 domain. *PLoS One* **7,** e33586 (2012).

6. Hertzog, M. & Carlier, M.-F. Functional characterization of proteins regulating actin assembly. *Curr. Protoc. Cell Biol.* **Chapter 13,** Unit 13.6 (2005).

7. Doolittle, L. K., Rosen, M. K. & Padrick, S. B. Measurement and analysis of in vitro actin polymerization. *Methods Mol. Biol.* **1046,** 273–293 (2013).
